# Supplementary material for: Perception, Knowledge, and Consumption Potential of Crude and Refined Palm Oil in Brazilian Regions
Source: Foods. 2024 Sep 15;13(18):2923. doi: 10.3390/foods13182923 (PMC11431150; doi:10.3390/foods13182923)

Perception, Knowledge, and Consumption Potential of Crude and Refined Palm Oil in Brazilian Regions

Alana Moreira Bispo[1]; Agnes Sophia Braga Alves[2]; Edilene Ferreira da Silva[3]; Fernanda Doring Krumreich[1]; Itaciara Larroza Nunes[3]; Camila Duarte Ferreira Ribeiro[1,2]*

[1] Nutrition School, Federal University of Bahia, Basílio da Gama Street, w/n-Campus Canela, Salvador, Bahia 40110-907, Brazil

[2] Graduate Program in Food Science, Faculty of Pharmacy, Federal University of Bahia, Campus Ondina, Salvador Bahia 40170-290, Brazil

[3] Graduate Program in Food Science, Department of Food Science and Technology, Federal University of Santa Catarina, Admar Gonzaga Highway, 1346, Itacorubi, Florianópolis, Santa Catarina 88034-000, Brazil

Figure S1 Arrangement of questions to analyze perception, cognition, preference, attitude, and consumption frequency of CPO and RPO

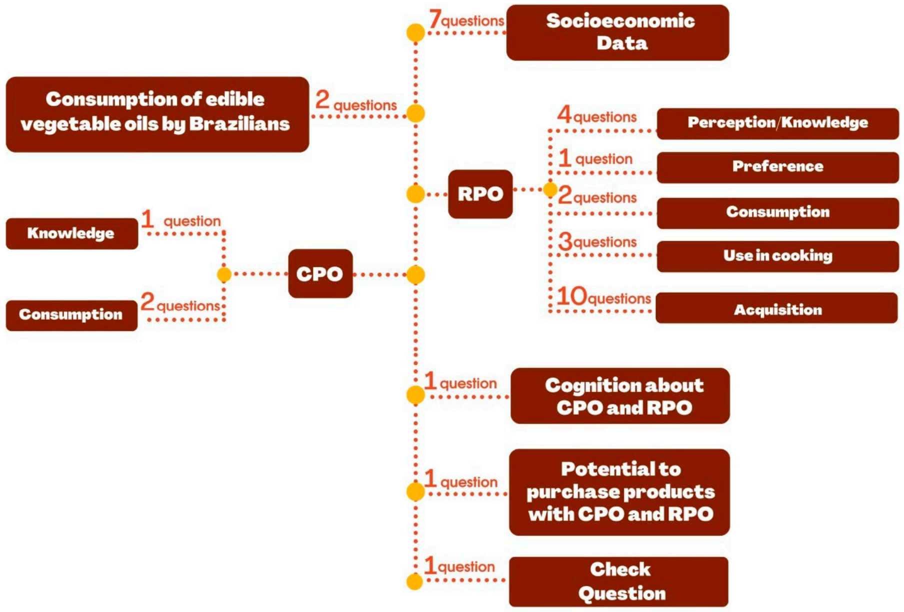

Figure S2 Most consumed oils or olive oils by respondents and the main reasons for their choice. Source: Author's own

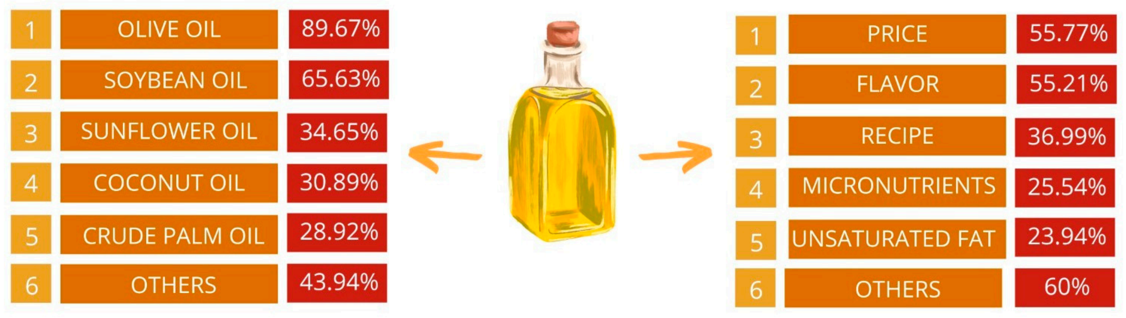

Note 1: Olive oils are extracted from the pulp of fruits, while oils are obtained from seeds, pits, or oleaginous grains (CARDOSO, 2006).

Note 2: In these questions, respondents could select all the alternatives they wished. Therefore, the sum of the percentages exceeded 100%.

Figure S3 Respondents' Knowledge about CPO

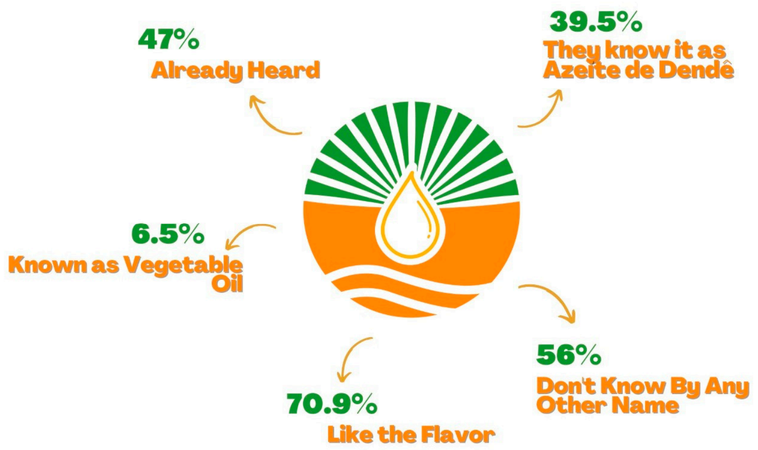

Figure S4 Respondents' Knowledge about CPO. Source: Author's own

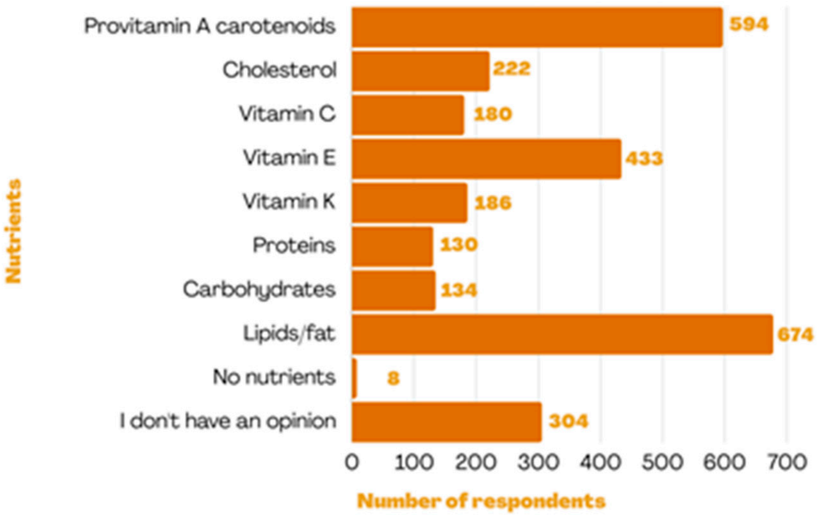

Note: Number of respondents in absolute values. In this question, participants could select all the alternatives they wished, hence the sum of percentages exceeding 100%.

Figure S5 Respondents' Knowledge about General Aspects of CPO

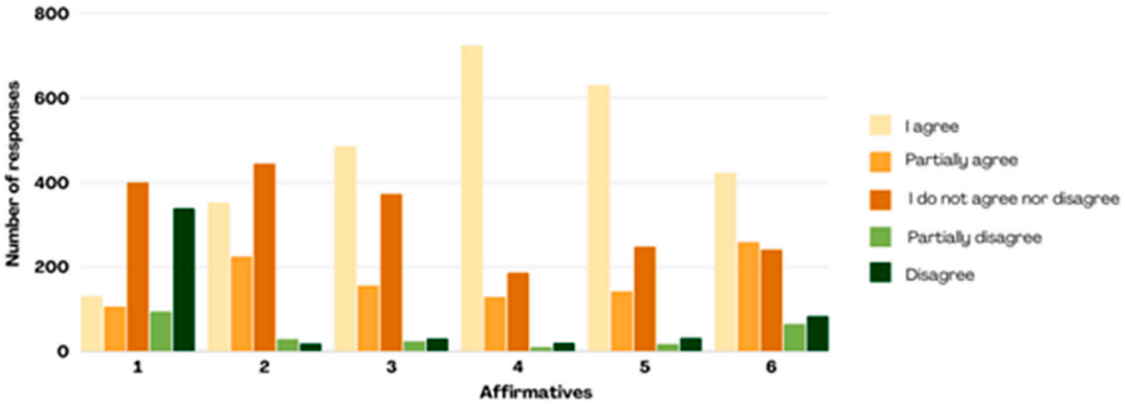

1 - It is the most produced vegetable oil in the world, surpassing the production of soybean oil.
2 - It is rich in antioxidant substances, which have been studied in the prevention of diseases such as cancer, Alzheimer's, and Parkinson's.
3 - It has two phases: one liquid and one solid.
4 - It is used in the preparation of dishes in African-derived religious ceremonies.
5 - It can be used in the preparation of dishes on Catholic religious holidays, such as Holy Week and the Day of Saints Cosmas and Damian.
6 - When choosing a food, I am concerned if its production is associated with deforestation and loss of biodiversity.

Figure S6 Comparison of the percentage of Crude Palm Oil purchase in the regions of Brazil

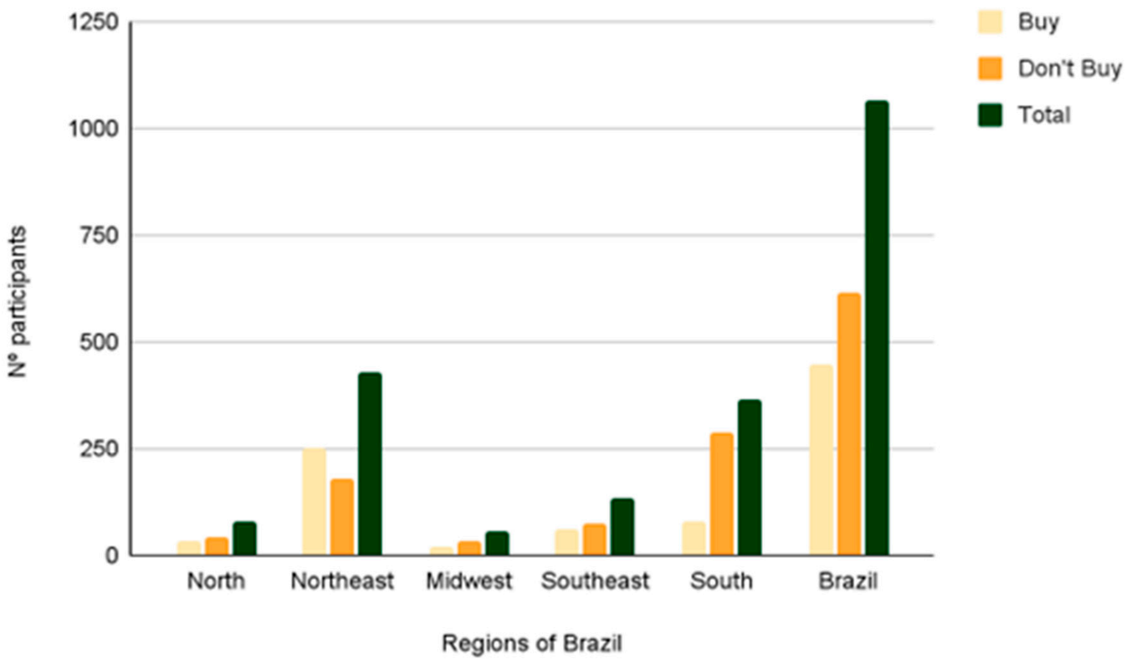

Figure S7 Percentage of purchase, acquisition methods, storage, and disposal of CPO by respondents

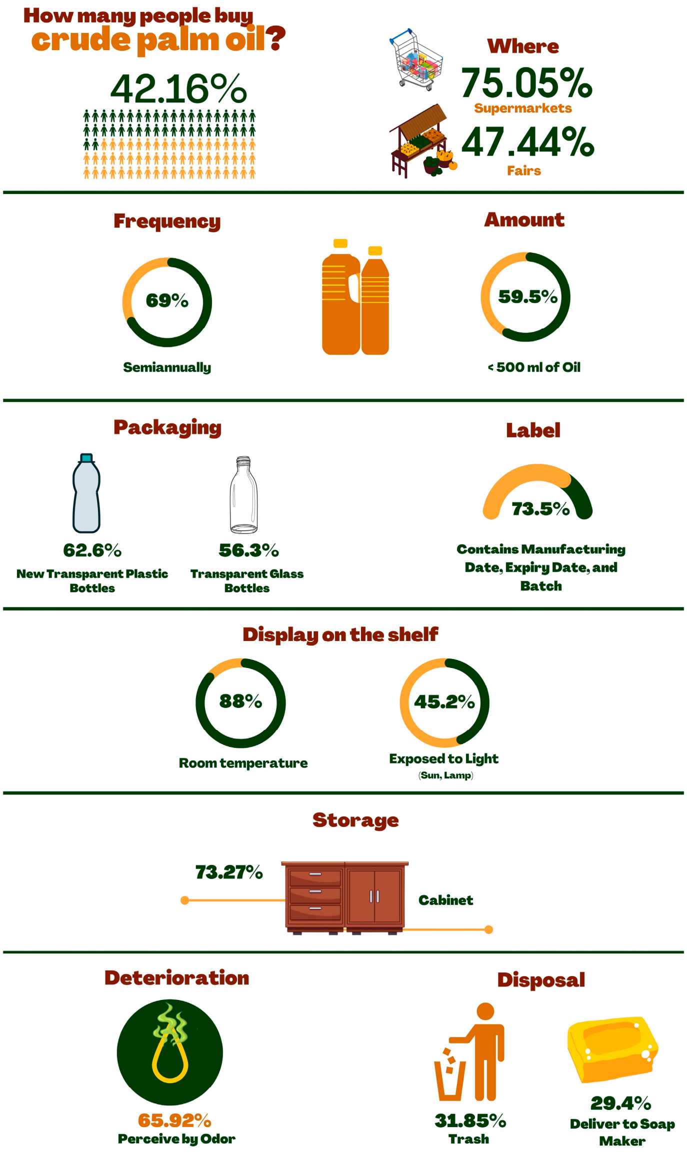

Figure S8 Consumption of foods prepared with CPO by respondents (a) and description of foods prepared with CPO (b)

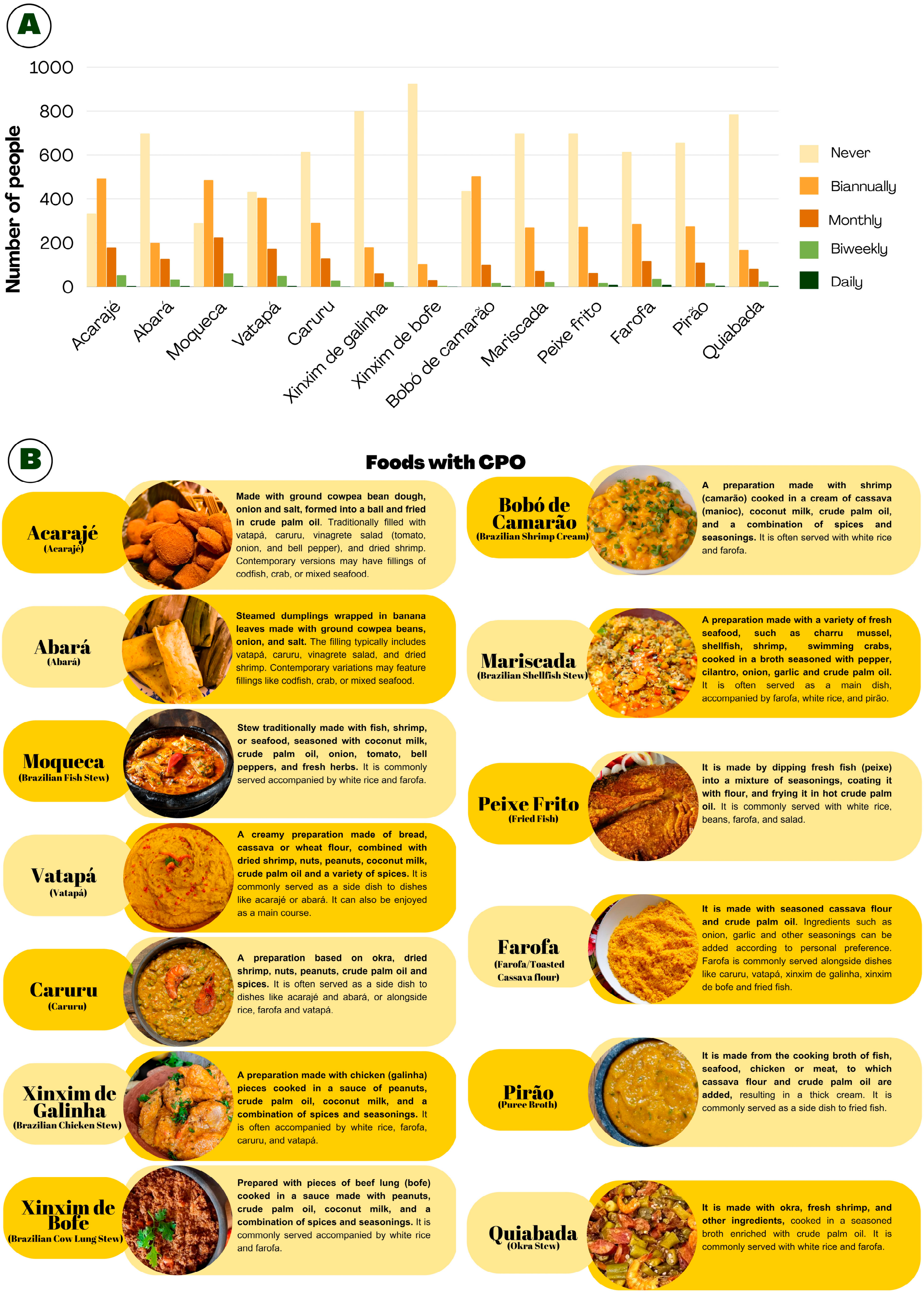

Figure S9 Knowledge of Respondents Regarding General Aspects of RPO

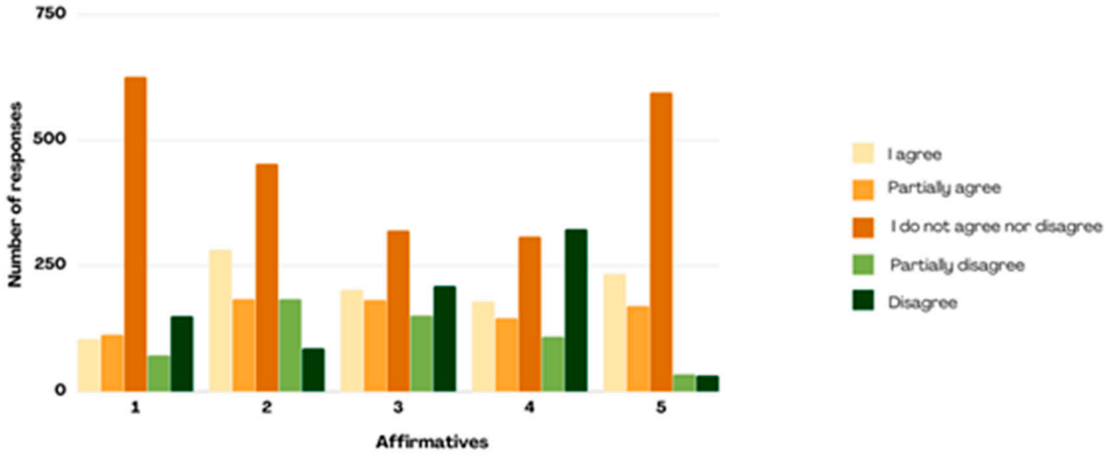

1 - In the refining process of palm oil, toxic substances are formed, which can cause cancer.

2 - Crude palm oil or palm kernel oil is less used in the food industry compared to refined palm oil.

3 - I dont mind if a product contains refined palm oil, as long as it tastes good.

4 - When I buy a processed product, I usually read its label to see if it contains refined palm oil.

5 - Refined palm oil is used in the industry as a substitute for trans fat or partially hydrogenated, hydrogenated, and/or interesterified vegetable fat.

Figure S10 Consumption of Foods with RPO by Respondents

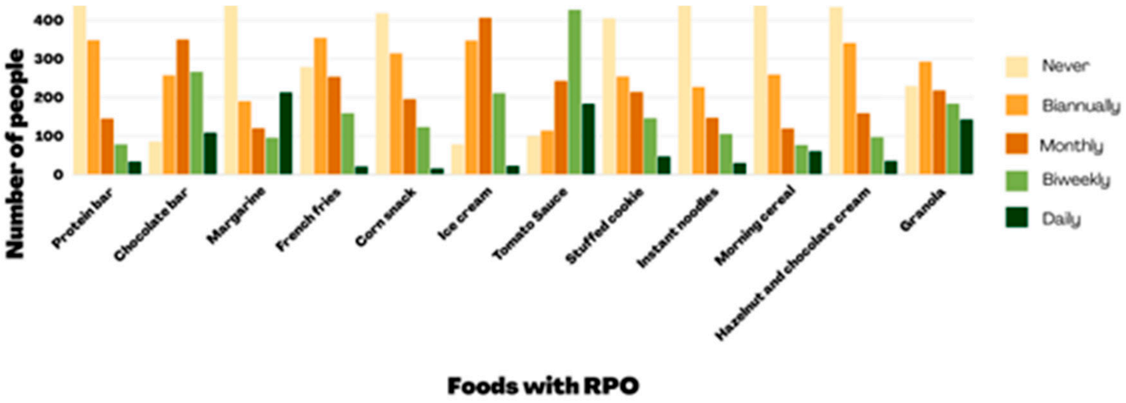

Supplement: Supplementary file 1 [file foods-13-02923-s001.zip › foods-3163744-supplementary.pdf]
